# Mechanical Properties of Recycled Concrete in Marine Environment

**DOI:** 10.1155/2013/728357

**Published:** 2013-05-07

**Authors:** Jianxiu Wang, Tianrong Huang, Xiaotian Liu, Pengcheng Wu, Zhiying Guo

**Affiliations:** ^1^College of Civil Engineering, Tongji University, Shanghai 200092, China; ^2^College of Marine Environment and Engineering, Shanghai Maritime University, Shanghai 200135, China; ^3^Shanghai International Shipping Service Center Development Co., Shanghai 200120, China

## Abstract

Experimental work was carried out to develop information about mechanical properties of recycled concrete (RC) in marine environment. By using the seawater and dry-wet circulation to simulate the marine environment, specimens of RC were tested with different replacement percentages of 0%, 30%, and 60% after immersing in seawater for 4, 8, 12, and 16 months, respectively. Based on the analysis of the stress-strain curves (SSCs) and compressive strength, it is revealed that RC' peak value and elastic modulus decreased with the increase of replacement percentage and corroding time in marine environment. And the failure of recycled concrete was speeded up with more obvious cracks and larger angles of 65° to 85° in the surface when compared with normal concrete. Finally, the grey model (GM) with equal time intervals was constructed to investigate the law of compressive strength of recycled concrete in marine environment, and it is found that the GM is accurate and feasible for the prediction of RC compressive strength in marine environment.

## 1. Introduction

The use of recycled materials as an aggregate in concrete has become popular recently in terms of reducing the consumption of natural aggregate and for the environmental advantage of the disposal of waste materials. To make this technology feasible, a significant amount of experimental works has been conducted. And it has proved that some properties of recycled concrete may be generally lower than those of normal concrete, but they are still sufficient for practical application in some constructions and buildings [1, 2]. 

The most important mechanical properties of recycled concrete are the compressive strength, the tensile and the flexural strengths, the bond strength, and elastic modulus of such concrete. For the peak value of e stress-strain curve that yields the compressive strength, several investigations have been performed for the stress-strain relation of recycled concrete in recent years. Bairagi et al. [3] discovered that similar trends existed in the stress-strain curves of recycled concrete, and the curvature of each curve gradually improved with the increase of replacement percentage. Topcu [4] found in his investigation that the values of compressive strength, toughness, plastic energy capacity and elastic energy, and the elastic modulus decrease with the increase of recycled coarse aggregated amount. Rqhl and Atkinson [5] reached a conclusion that the peak strain increases as the recycled aggregates increase after they investigated the complete stress-strain curve of recycled concrete with different replacement content. Xiao et al. [6] found that the failure mode of recycled aggregate concrete is a shear mode, and the replacement percentage has a considerable influence on the stress-strain curves of recycled aggregate concrete. After a series of tests, Adom-Asamoah and Afrifa [7] discovered that the trends in the development of compressive and bending strengths of plain phyllite concrete were similar to those of conventional concrete, but the compressive and bending strengths of phyllite concrete mixes were on the average 15–20% lower than those of the corresponding granite concrete mixes. Somna et al. [8] learned that the modulus of elasticity of recycled aggregate concrete with and without ground bagasse ash was lower than that of conventional concrete by approximately 19% after their investigation. However, there are few reports which were concerned about the mechanical properties of recycled concrete in marine environment until now, which prohibits a wider application of recycled concrete in the practical design of civil engineering structures.

In this study, experiments are conducted to provide a comprehensive analytical evaluation of the mechanical properties of RC in marine environment. The results are compared with normal concrete in normal environment. The stress-strain relations of RC with different replacement content and corroding time are explicitly analyzed, and the elastic modulus and failure behaviors are also included. And a grey model (GM) with equal time intervals was constructed to investigate the decaying law of RC compressive strength in marine environment, by which the durability of RC in marine environment was revealed. The results presented in this paper are significant to efficiently use recycled concrete in practical engineering in marine environment.

## 2. Experimental Descriptions

### 2.1. Materials

Ordinary Portland cement with 28d compressive strength of 42.5 MPa was used in this research. And the fine aggregate used was river sand with a fineness modulus of 2.8. The coarse aggregate includes natural coarse aggregate (NCA) and recycled coarse aggregate (RCA) obtained from the building demolition in Pudong Avenue, Shanghai, China. Their physical properties are shown in Table 1.

### 2.2. Mix Proportions

 Ye et al. [9] suggest that common mixing methods are only fit for the recycled concrete C25 and the lower strength grades in China (C25 represent that the compressive strengths is not less than 25 MPa after 28 days of standard curing). Therefore, this research was using the C25 mix proportion, and the water/cement ratio was kept constant as 0.55. The mixture was divided into three groups. The main difference between these three groups is the replacement percentage, which is 0%, 30%, and 60%, respectively. The case of replacement percentage 0% is normal concrete, which serve as the reference concrete. The mix proportions of concretes are shown in Table 2.

### 2.3. Preparation of Specimens

The preparations of specimens were performed in the Laboratory for Concrete Material Research at Shanghai Maritime University in Shanghai, China. All mixings were conducted under laboratory conditions. The sand, cement, and coarse aggregates were placed and dry mixed for about 2 minutes before water was added. After 3 minutes of mixing followed water was added, a slump test was run to determine its workability. The mixture in each group was cast in 100 × 100 × 100 mm cubes in three steel moulds and then compacted on vibration table. They were demolded a day after casting and were cured in a fog room (20 ± 2°C,  95% relative humidity) for 28 days. The cube specimens were used to obtain the cube compressive strength of the RAC.

After 28-day curing, all specimens are submerged in the seawater for 8 hours and out for 16 hours every day, which is to simulate the tidal zone in marine environment. The duration of this dry-wet circulation lasted for 4, 8, 12, and 16 months, respectively. The specimens were cleaned up and dried naturally when the circulation ended. And each group to be tested includes three specimens in the same curing condition. The seawater used was picked up from the Huang-hai Sea, which is one of the four key seas of China. Its content is listed in Table 3 [10]. 

### 2.4. Test Setup and Test Method

**Figure 1:**
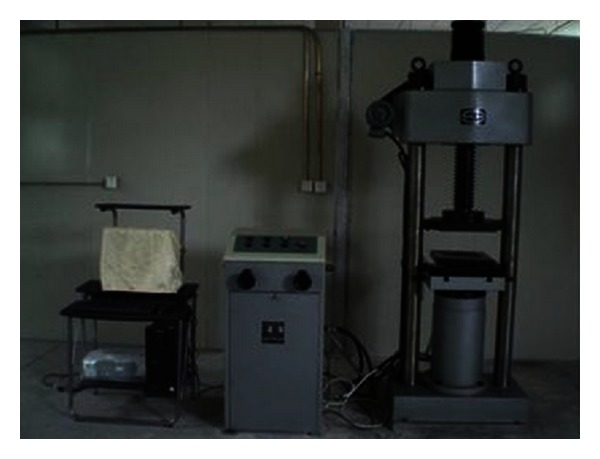
Test setup.

The loading setup as shown in Figure 1 was microcomputer-controlled electrohydraulic servotester. During the experiment, the axial compression and the vertical deformation of the test specimens were automatically collected by the computer installed. The measured deformation is the deformation of the top of the specimen. Each specimen was preloaded before the actual loading in order to lessen the impacts on the test results due to the loose of the specimen end. While preloading, 30–40% of the estimated peak loading (based on the test results for the cube compressive strength) is applied, and the loading is repeated three times.

## 3. Test Results and Discussion

### 3.1. Stress-Strain Curves

**Figure 2:**
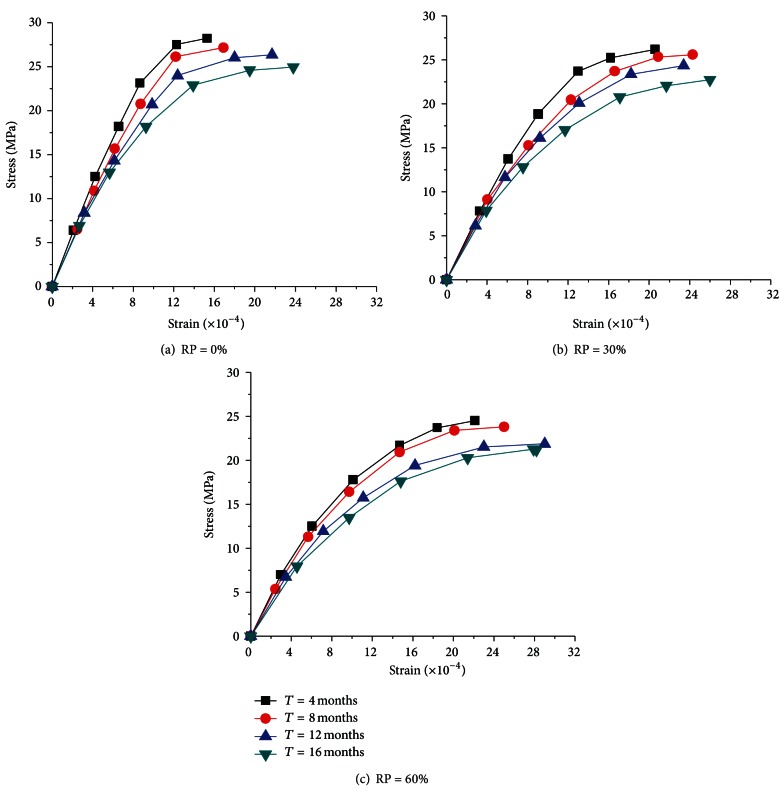
Typical stress-strain curves of RC in marine environment.

The typical stress-strain curves (SSCs) of RC in marine environment with different RCA contents and corroding time are shown in Figure 2.

Figure 2 illustrates that the shape of the stress-strain curve for all the RCs in marine environment was similar to that of the natural aggregate concrete, irrespective of the RCA replacement percentage and corroding time, which leads to the conclusion that the theory of plasticity would be still suitable for structural design process. Roughly speaking, the SSC includes three characteristic parts. The first part is the linear portion; the second part is the nonlinear portion of the ascending branch, and the third part represents the descending branch.

Another notable fact of the SSC is that the RCA replacement percentage and corroding time have remarkable influences on the SSC of RC. The ultimate stress of the SSC decreases with the increasing amount of replacement percentage. And the ultimate stress of the SSC decreases with the corroding time for the same series, which means that the compressive strength decreases with the corroding time. This also demonstrates that the plastic deformation and residual strength of RC decreases, and the destruction process accelerates with the increase of replacement percentage and corroding time. 

### 3.2. Compressive Strength

**Figure 3:**
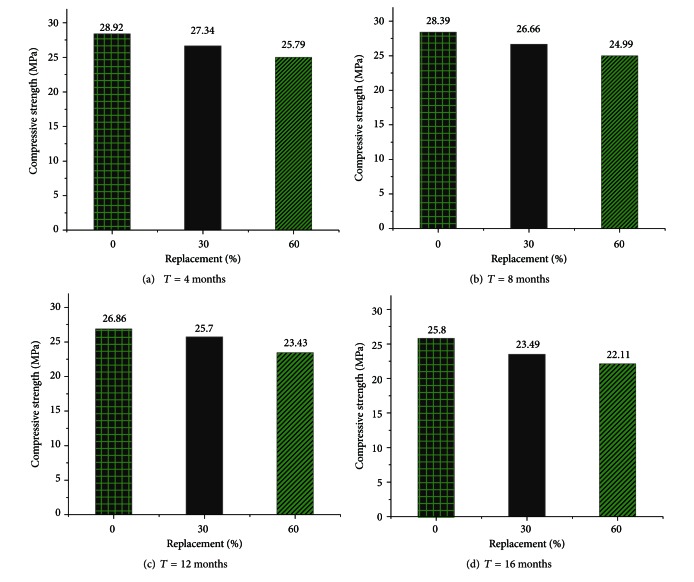
Compressive strength of RC in marine environment.

 Based on the test procedure in 2.3, every group has three peak values, and its mean value yields the compressive strength. The results of all tests are shown in Figure 3.

Figure 3 indicates that the compressive strength of RC decreases with the replacement percentage. The decreasing range of compressive strength is 1.16 to 2.31 MPa at different corroding time when the replacement percentage increases from 0% to 30%. And this decreasing range goes up from 3.13 to 3.69 MPa when the replacement percentage increases from 0% to 60%. Because the NC is normal concrete and RC-30 is recycled concrete with its replacement percentage as 30%, it can be found that the suitable replacement percentage should not exceed 30% in marine environment, which agreed with those obtained by Kasai [11].

Meanwhile, the compressive strength of RC decreases gradually as the corroding time increases. It presents the process of the decreasing of RC in marine environment quantitatively in Table 4.

From Table 4, it can be seen that the compressive strength decreases at about 2% and not less than 25 MPa when the corroding time is within 8 months. And when the time increases to 12 months and 16 months, the compressive strength becomes smaller than 25 MPa with a decreasing range of 4% to 8%. It can be interpreted that the destruction process accelerates after internal bonding is gradually destroyed. Therefore, the corroding of seawater cost, time, and it has promising future for the application of recycled concrete.

### 3.3. Modulus of Elasticity

The elastic modulus *E*_*c*_ of the recycled concrete was determined from compressive strength by the following empirical equation [12]:

(1) $$\begin{array}{c} E_{C}=5.639\sqrt{f_{c}}-4.952, \end{array}$$

where *E*_*C*_ represents the modulus of elasticity of concrete (GPa); *f*_*c*_ is the compressive strength of concrete (MPa). 

**Figure 4:**
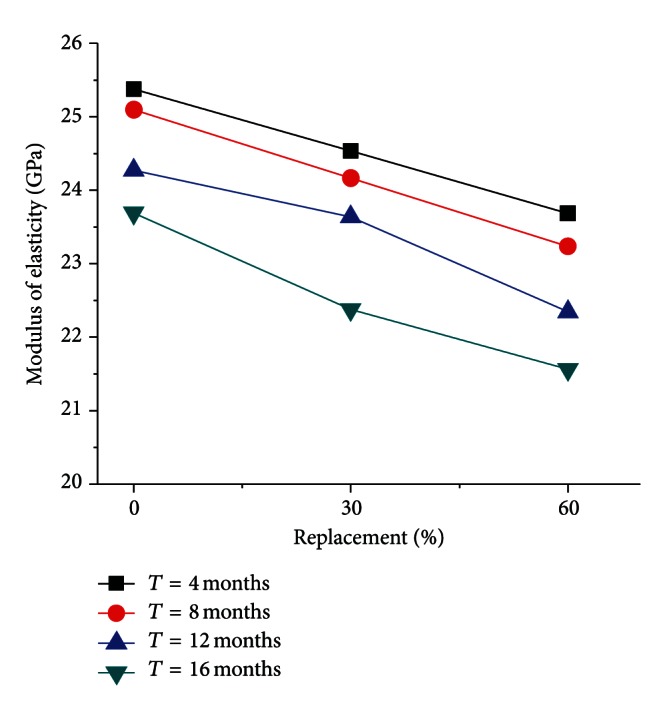
Elastic modulus with different RP.

**Figure 5:**
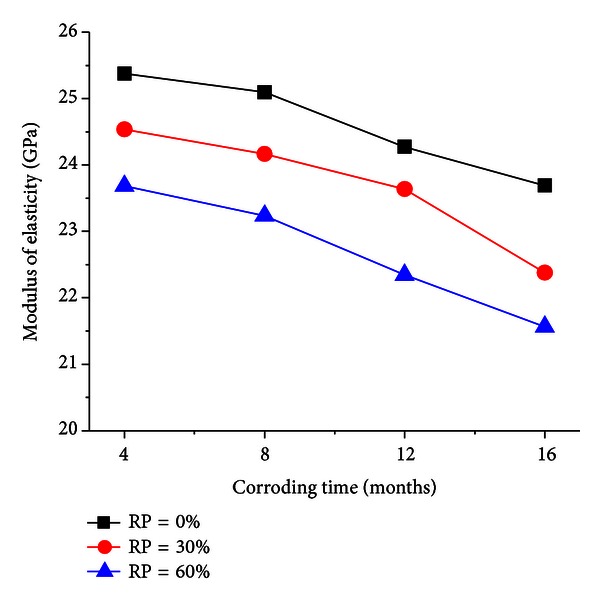
Elastic modulus with different CT.

The elastic modulus of RC is shown in Figure 4, versus different replacement percentage, and Figure 5, versus different corroding time (*T* and RP represent corroding time and replacement percentage, resp.). 

Figure 4 shows that the elastic modulus of the RC in marine environment is lower than that of normal concrete (i.e., RP = 0%), and it decreases with increasing replacement percentage. When the replacement percentage is 60%, the elastic modulus is reduced by about 7.5%. This is caused by the application of the RAC with a lower elastic modulus than that of the natural coarse aggregate.

 And Figure 5 indicates that the corroding of seawater has considerable influence on the elastic modulus of the RC. The elastic modulus of the RC in marine environment is decreasing with the corroding time, and its decreasing accelerates when the corroding time increases. When the corroding time is 8 months, the elastic modulus drops by 2%, but the elastic modulus is reduced by 9% when the corroding time increases to 16 months. Thus, it can be seen that the elastic modulus decreases gradually in marine environment as the seawater penetrates into the inner of recycled concrete.

### 3.4. Failure Behavior

#### 3.4.1. Normal Concrete

 In the early stage of loading, the test specimens did not show any cracks. As the compression loading increases, small vertical microcracks were gradually forming in the surface of test specimens. When reaching the peak stress, several discontinuous short vertical cracks appeared, and they combined into inclined macrocracks in the end, which means the destruction of the concrete. The inclination angle of the macrocracks with respect to the vertical loading plumb is about 59–62°, which fits well with the research of normal concrete in common environment by Xiao et al. [6].

#### 3.4.2. Recycled Concrete

**Figure 6:**
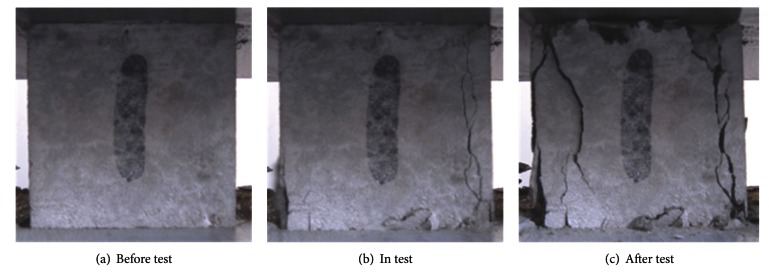
Failure process of recycled concrete in marine environment.

 As in the normal concretes the specimens of RC did not show any cracks in the early loading stage. However, very short ad thin vertical microcracks appeared when the compression loading exceeded the peak value. By continuing the test, an inclined macrocrack was formed quickly through the specimen, and the load went down in an instance. Sound of cracking was heard for some samples. And some vertical or slightly inclined branch cracks could be seen on some specimens when the loading keeps constant. All the test specimens displayed an inclined failure plane, with an inclination angle of about 65–85° with respect to the loading plumb. The inclination angle of the failure plane of RC in marine environment is not only significantly larger than that of the normal concrete; but also larger than recycled concrete in normal condition. Therefore, the plastic deformation of RC in marine environment is less than that of normal concrete and concrete in normal condition. The typical failure process of RC in marine environment is shown in Figure 6.

## 4. Application of Grey Model (GM) in Prediction

### 4.1. Information about GM

Grey model (GM) was developed by Professor Deng in the 1980s. The basic idea of the grey theory is like this. (1) Measured data are processed by accumulation so that the influence of the stochastic factor in the sequence of the data is decreased, and the internal law in the sequence of the data becomes obvious. (2) The sequence of the data becomes a variable in the grey model, which has a combination of the differential, difference, and approximate index [12]. The steps for building the grey model are given in the appendix.

The grey theory does not require a large amount of data and the typical distributing order, but it has high prediction precision; thus, it is widely used for prediction in agriculture, geology, and civil engineering, and so forth [13–17]. All the results indicate that the grey theory can predict reasonably well compared to the measured value, and the difference is in an acceptable range.

The decaying of compressive strength of RC is a complex process in marine environment, which is affected by many factors. This process cannot be explicitly expressed by a mathematical formula. But it is not totally unknown like a “black box” whose internal structure, parameters, and characteristics are unknown. The grey theory model is applicable under these conditions and had been successfully used for prediction of compressive strength of normal concrete in seawater environment in 2004 by Lin and others [18]. However, few researchers have used GM to predict compressive strength of recycled concrete in marine environment up to now.

### 4.2. Constitution of GM and Prediction

Based on test results of compressive strength, the data used to build the GM is listed in Table 5.

According to Table 5 and the steps of the appendix, the grey model for NC, RC-30, and RC-60 is constructed as follows, respectively:

(2) $$\begin{array}{c} {\hat{x}}_{{\left(k+1\right)}_{A}}^{\left(1\right)}=-1312.84113\exp\left(-0.02219k\right)+1341.7611,\\ {\hat{x}}_{{\left(k+1\right)}_{B}}^{\left(1\right)}=-1036.1320\exp\left(-0.02675k\right)+1063.4687,\\ {\hat{x}}_{{\left(k+1\right)}_{C}}^{\left(1\right)}=-906.8300\exp\left(-0.02858k\right)+932.6167. \end{array}$$

The prediction value is obtained by the following equation:

(3) $$\begin{array}{c} {\hat{x}}_{\left(k\right)}^{\left(0\right)}={\hat{x}}_{\left(k\right)}^{\left(1\right)}-{\hat{x}}_{\left(k-1\right)}^{\left(1\right)}\;\left(k=2,3,\ldots ,N\right). \end{array}$$

By using the above equations, predicted value of compressive strength in marine environment was acquired, and the comparison of test value and predicted value was listed in Table 6.

**Figure 7:**
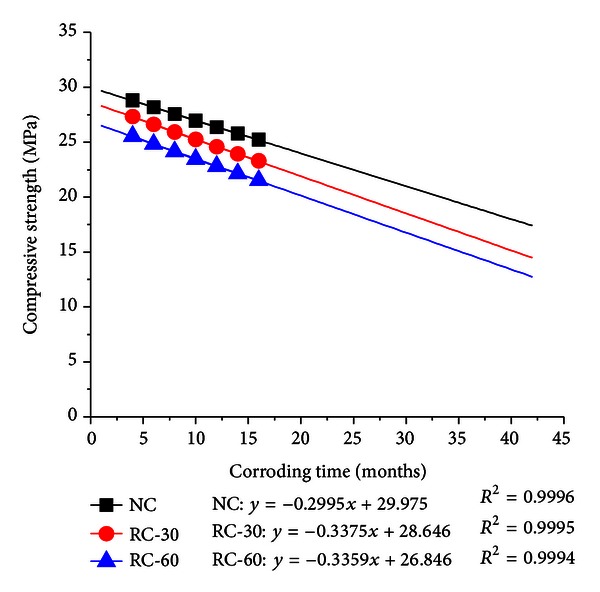
Regressive fitting of RC compressive strength in marine environment.

Table 6 shows that predicted value is well confirming to the test value, which demonstrates that grey model is feasible for the prediction of compressive strength of recycled concrete in marine environment. The predicted value is very similar to the test value with a low relative error below 5%. In order to know long-term effect of seawater, the predicted GM value was also applied to draw regressive equation; thus, the law of decaying of compressive strength of RC in marine environment was illustrated in Figure 7 (*R* ^2^ represents the correlation coefficient).

Figure 7 shows that the compressive strength of RC decreases with corroding time in marine environment linearly. Its slope is about −0.29 to −0.33. The compressive strength of RC is decreasing coherently, which is also testifying to the validity of the test results. According to the regressive equation, it was found that the compressive strength of NC, RC-30, and RC-60 drops by 33.2%, 38.2%, 40.8%, respectively, after 40-month corroding in marine of environment. This has reminded us that prevention measures should be taken to improve the durability of recycled concrete in marine environment.

## 5. Conclusions

In this paper, test results for the mechanical properties of recycled concrete in marine environment are presented and discussed. From this investigation, the conclusions can be drawn as follows.

1. The shape of the stress-strain curve for all the recycled concrete in marine environment was similar to that of the natural aggregate concrete, irrespective of the RCA replacement percentage and corroding time. However, the RCA replacement percentage and corroding time have remarkable influences on the SSC of RC.
2. The compressive strength of RC decreases with the replacement percentage, and the suitable replacement percentage should not exceed 30% in marine environment.
3. The compressive strength of RC also decreases with the corroding time. It decreases at 2% when the corroding time is within 8 months, and it decreases from 4% to 8% when corroding time exceeds 8 months.
4. The elastic modulus of the RC in marine environment is lower than that of normal concrete, and it decreases with increasing replacement percentage and corroding time. The elastic modulus is reduced by about 7.5% when the replacement percentage is 60%, and it dropped by 2% and 9% when the corroding time is within or over 8 months, respectively.
5. The failure behavior of normal concrete in marine environment is similar to that in common environment. But the failure process of RC in marine environment takes less time with a larger inclination angle of 65–85° between the failure plane and the loading plumb.
6. The grey model is feasible for the prediction of RC compressive strength in marine environment. Its accuracy is very high with a relative error less than 5%. The regressive equation shows that the compressive strength of NC, RC-30, and RC-60 drop by 33.2%, 38.2%, 40.8%, respectively, after 40-month-corroding in marine of environment.

## Figures and Tables

**Table 1:** Physical properties of NCA and RCA.

Physical index	NCA	RCA
Grading (mm)	5–32.5	5–32.5
Bulk density (kg/m^3^)	1466	1305
Apparent density (kg/m^3^)	2812	2498
Water absorption (%)	0.45	9.15
Crush index (%)	4.12	14.9

**Table 2:** Mix proportions of concretes (kg/m^3^).

No.	Replacement percentage	Water/cement	Cement	Sand	NCA	RCA	Mixing water
NC	0	0.55	425	520	1305	—	234
RC-30	30	0.55	425	500	874	375	234
RC-60	60	0.55	425	480	496	745	234

**Table 3:** Content of seawater (g/L).

Content	NaCl	MgCl_2_	MgSO_4_	CaSO_4_	K_2_SO_4_	CaCO_3_
Amount	27.2	3.8	1.7	1.2	0.9	0.1

**Table 4:** Decreasing of compressive strength with corroding time.

Samples	Compressive strength (MPa) (%)		
	NC	RC-30	RC-60
4 months	28.92 (100)	27.34 (100)	25.79 (100)
8 months	28.39 (98)	26.66 (98)	24.99 (98)
12 months	26.86 (93)	25.70 (94)	23.43 (91)
16 months	25.80 (89)	23.49 (86)	22.11 (86)

**Table 5:** Data for building the grey model (MPa).

No.	*K*_1_	*K*_2_*	*K*_3_	*K*_4_*	*K*_5_	*K*_6_*	*K*_7_
NC	28.92	28.65	28.39	27.62	26.86	26.33	25.80
RC-30	27.34	27.00	26.66	26.18	25.70	24.60	23.49
RC-60	25.79	25.39	24.99	24.21	23.43	22.77	22.11

*Is the mean value of *k*_*i*−1_ and *k*_*i*+1_ (*i* = 2, 4, 6).

**Table 6:** Comparison of test value and predicted values.

No.	Corroding time (months)	Measured value (MPa)	Predicted value (MPa)	Relative error (%)
NC	4	28.92	28.81	0.38
	6	28.65	28.18	1.67
	8	28.39	27.56	2.92
	10	27.62	26.95	2.43
	12	26.86	26.36	1.86
	14	26.33	25.78	2.08
	16	25.80	25.22	2.25

RC-30	4	27.34	27.34	0
	6	27.00	26.62	1.41
	8	26.66	25.92	2.78
	10	26.18	25.24	3.59
	12	25.70	24.57	4.39
	14	24.60	23.92	2.72
	16	23.49	23.29	0.85

RC-60	4	25.79	25.55	0.93
	6	25.39	24.83	2.2
	8	24.99	24.13	3.44
	10	24.21	23.45	3.14
	12	23.43	22.79	2.73
	14	22.77	22.14	2.72
	16	22.11	21.52	2.67

## Appendix

### Constitution of Grey Model

Let the sequence of initial time-interval data be:

(A.1) $$\begin{array}{c} x^{\left[0\right]}=\{x^{\left(0\right)}\left(t_{1}\right),x^{\left(0\right)}\left(t_{2}\right),\ldots ,x^{\left(0\right)}\left(t_{n}\right)\} \end{array}$$

with time step *t*_*i*+1_ − *t*_*i*_ = *k* = constant and *i* = 1,…, *n* − 1.

By accumulative addition, a smooth sequence is obtained:

(A.2) $$\begin{array}{c} x^{\left[1\right]}=\{x^{\left(1\right)}\left(1\right),x^{\left(1\right)}\left(2\right),\ldots ,x^{\left(1\right)}\left(n\right)\}, \end{array}$$

where *x* ^[1]^(*k*) = ∑_*i*=1_^*k*^ *x* ^(1)^(*t*_*i*_).

Based on the above expression, we can obtain the differential equation

(A.3) $$\begin{array}{c} \frac{dx^{\left(1\right)}}{dt}+ax^{\left(1\right)}=c, \end{array}$$

where *c* = ∑_*i*=1_^*n*−1^ *b*_*i*_ *x* ^(1)^(*i* + 1) and the denoting coefficient vector $\hat{a}={\left[a,c\right]}^{T}$.

The solution is

(A.4) $$\begin{array}{c} {\hat{x}}^{\left(1\right)}\left(k+1\right)=\left[x^{\left(0\right)}\left(t_{1}\right)-\frac{c}{a}\right]\exp\left(-ak\right)+\frac{c}{a}, \end{array}$$

where coefficients *a* and *c* are the parameters to be identified and can be obtained by the least square method.
